# ASlive: a database for alternative splicing atlas in livestock animals

**DOI:** 10.1186/s12864-020-6472-9

**Published:** 2020-01-30

**Authors:** Jinding Liu, Suxu Tan, Shuiqing Huang, Wen Huang

**Affiliations:** 1 College of Information Science and Technology, Nanjing Agricultural University, Nanjing, 210095 China; 2 Research Center for Correlation of Domain Knowledge, Nanjing Agricultural University, Nanjing, 210095 China; 3 Department of Animal Science, Michigan State University, East Lansing, MI 48824 USA

## Abstract

**Background:**

Alternative splicing is an important biological process whose precision must be tightly regulated during growth and development. Although there are species, disease (e.g. cancers), or study specific databases available in many organisms, no database exists in livestock animals specifically tailored for alternative splicing.

**Description:**

We present in this study the development and implementation of a database for alternative splicing atlas in livestock animals (ASlive.org). Using publicly available RNASeq data sets across many tissues, cell types, and biological conditions totaling 28.6 T bases, we built a database of alternative splicing events in five major livestock and poultry animal species (cattle, sheep, pigs, horses, and chickens). The database contains many types of information on alternative splicing events, including basic information such as genomic locations, genes, and event types, quantitative measurements of alternative splicing in the form of percent spliced in (PSI), overlap with known DNA variants, as well as orthologous events across different lineage groups.

**Conclusions:**

This database, the first of its kind in livestock animals, will provide a useful exploratory tool to assist functional annotation of animal genomes.

## Background

Splicing of multi-exonic precursor messenger RNAs (pre-mRNAs) is a key biological process that can impact both the sequences and expression of proteins. In particular, multi-exonic pre-mRNAs have the potential to be alternatively spliced. Alternative splicing allows one gene to code for multiple mature mRNA and protein isoforms, greatly expanding the diversity of the proteome [1]. For example, the Drosophila Down syndrome cell adhesion molecule (*Dscam*) gene is able to generate more than 38,000 possible isoforms with variable immunoglobin and transmembrane domains [2]. This remarkable diversity of a transmembrane receptor gene provides the specificity for neuronal connectivity needed in axon guidance. The precise regulation of alternative splicing is important in development and growth. Thus, the disruption of normal alternative splicing can lead to diseases such as cancers. Indeed, natural DNA variation that results in genetic variation in alternative splicing is a major determinant of phenotypic diversity among individuals in a population, including genetic risks to diseases [3]. In livestock animals, where genetic improvement is a major goal, the specific role of alternative splicing in determining phenotypic variation in economic traits is not well understood. Part of the reason is the lack of a comprehensive annotation of alternative splicing in these agricultural species. For example, while the size of the genome (3.1 Gbp for humans and 2.7 Gbp for cattle) and number of protein coding genes (20,454 for humans and 21,880 for cattle) are similar for humans and cattle, there are on average 5.1 annotated splice isoforms per human gene versus 1.6 per cattle gene, a more than three-fold difference [4].

The advent of high throughput sequencing technologies has greatly facilitated genome annotation efforts. In addition, targeted experimental studies have increasingly utilized next generation sequencing to globally survey the transcriptomes of different cell types, tissues, and animals across many organisms. Such diversity of experimental data provides unprecedented breadth and depth of transcriptomes across many species in public databases, including livestock animals. However, most studies focus on differences in steady state RNA abundance, which represents an equilibrium between transcription and mRNA decay and does not capture difference in post-transcriptional regulation such as splicing.

Experimental data in public databases such as the sequence read archive (SRA) are highly heterogeneous. While this presents a challenge to re-use these data, it also provides a great opportunity to discover new information, some of which only happens in specific conditions. As such, heterogeneous and diverse experimental data in public databases complement organized annotation projects that typically only use limited samples and conditions. For example, even for humans, experimental data in the SRA database contained a large number of unannotated splice junctions [5].

There are several alternative splicing specific databases available. For example, the VastDB (vertebrate alternative splicing and transcription database) provides a comprehensive catalog of alternative splicing events in vertebrate animals compiled from a large number of publicly available RNA-Seq experiments [6]. The ASpedia (Alternative Splicing Encyclopedia of Human) database contains a collection of alternative splicing events identified from a single project with 26 tissues and 241 samples [7]. The CancerSplicingQTL is a database to search and browse splicing quantitative trait loci (sQTLs) affecting alternative splicing in cancer samples [8]. These databases become increasingly useful as an exploratory and hypothesis generating tool. However, no database is specifically designed for livestock animals.

In this study, we present the development of the alternative splicing in livestock animals (ASlive.org) and a web interface for users to interact with the database. There are several unique features of the database. We developed a uniform processing pipeline to process over 4000 samples in the SRA database, covering 188 tissues in five major livestock animal species (cattle, sheep, pigs, horses, and chicken), totaling 28.6 T bases of sequence data. We discovered hundreds of thousands of unannotated alternative splicing events that were supported by multiple lines of experimental evidence and quantitatively estimated their alternative splicing level. We also identified conserved alternative splicing events across species, allowing users to assess and explore the tissue and species specificity of alternative splicing events. This study provides an important new tool to the animal genome research community and complements ongoing large-scale annotation projects such as the functional annotation of animal genomes (FAANG) project [9].

## Construction and content

### Data collection

The reference genome assemblies of five livestock species including cattle (taxonomy id: 9913), sheep (9940), pigs (9823), horses (9796) and chicken (9031) were downloaded from Ensembl (release 96). We also obtained reference annotations from both Ensembl and RefSeq. Sequence data from a total of 4166 RNASeq experiments containing 8257 runs and 28.6 T bases in the SRA database were collected by querying the meta data of the SRA database (Table 1). To simplify our data processing pipeline, we restricted data to the Illumina platform, which constituted the vast majority of RNASeq data.

**Table 1:** Summary of RNASeq data used in ASlive

Species	Studies	Experiments	Runs	Tissues	Spots (Million)	Data volume (Tera bases)
Cattle	104	1443	2220	81	60,067	8.3
Sheep	32	708	3540	63	30,490	6.6
Pig	77	821	1133	65	31,864	5.9
Horse	20	317	317	18	9214	1.2
Chicken	109	877	1047	76	40,304	6.6
Total	334	4166	8257	188	171,939	28.6

### Improvement of gene models

The reference annotations from Ensembl and RefSeq were largely incomplete for livestock species. We used the following procedure to improve the annotations using high quality RNASeq data from SRA (Table 2).

**Table 2:** Summary of improvement of gene models

Species	Genome assembly	Ensembl+RefSeq			SRA data used	After improvement		
		Genes	Transcripts	Transcripts per gene	Total sequenced fragments (M)	Genes	Transcripts	Transcripts per gene
Cattle	ARS-UCD1.2	32,731	95,018	2.9	17,444	35,661	175,198	4.9
Sheep	Oar_v3.1	27,829	44,398	1.6	10,212	28,974	65,191	2.2
Pig	Sscrofa11.1	30,284	101,216	3.3	8982	31,959	157,045	4.9
Horse	EquCab3.0	35,886	111,890	3.1	2606	36,310	124,270	3.4
Chicken	GRCg6a	27,251	81,909	3.0	16,183	29,091	156,429	5.4

1. Ensembl and RefSeq annotations were compared using cuffcompare by setting Ensembl as the reference. RefSeq transcripts that were flagged as “j” (novel isoform) and “u” (novel transcribed region) were added to the Ensembl annotation. This merged annotation served as the reference annotation in subsequent steps.
2. Experiments with at least 40 million spots (30 million for horses due to low number of experiments passing the filter) and 75 bp read length were mapped to the reference genome using HISAT2 [10] in the presence of the reference annotation. Those with at least 40 million mapped fragments were retained and assembled into reference guided gene models in GTF format using StringTie [11].
3. We then improved the reference annotation by iteratively comparing each assembled GTF file to the annotation from the previous iteration. Briefly, one assembled GTF file was compared with the GTF file from the previous iteration using cuffcompare. Novel multi-exonic transcripts (“j” and “u”) that were at least 200 bp long, with an average coverage of 2x per transcript, and an average coverage of 1x per exon for all exons were added. This process was iteratively performed through all StringTie assembled GTFs from the previous step.
4. The final filtering step consisted of comparing all GTF files from step 2) to the merged GTF file from step 3) and requiring that all novel transcripts must occur in at least three different studies and four different experiments. All GTF files are available for download on the database website under the Summary page.

### Identification and quantification of alternative splicing events

After aligning RNASeq reads to the improved reference annotation in each species using HISAT2, we used rMATs [12] to identify and quantify alternative splicing events in all samples. rMATs reports junction read counts, effective junction length for each alternative splicing event and classifies them into five classes including alternative 5′ splice site (A5SS), skipped exon (SE), mutually exclusive exons (MXE), retained intron (RI), and alternative 3′ splice site (A3SS). It is important to note that rMATs is highly sensitive and does not rely on the GTF annotation to identify alternative splicing events and may report events that do not conform to existing intron chains in the annotation. We retained these events in our database because they were supported by junction reads. Alternative splicing events from all samples were merged to create a non-redundant catalog. To further refine the catalog, we retained events that were evident by at least three skipping reads and three inclusion reads in at least four different experiments and three different studies (Table 3). We identified between 48,208 and 151,087 confident alternative splicing events in each of the five species (Table 3). Quantitative measurements including the percent spliced in (PSI), numbers of skipping and inclusion reads, and the effective junction lengths were collected.

**Table 3:** Summary of alternative splicing events identified from SRA data

Species	A5SS	SE	MXE	RI	A3SS	Total
Cattle	10,227	82,153	25,130	20,364	13,213	151,087
Sheep	1567	50,030	11,148	2449	2390	67,584
Pig	8652	68,309	23,876	17,723	11,107	129,667
Horse	3176	29,564	6164	4358	4946	48,208
Chicken	10,088	58,752	19,415	19,892	12,128	120,275

### Identification of orthologous alternative splicing events

To enable comparative analyses, we first identified alternative splicing events that are orthologous among the livestock species. All alternative splicing events including those without sufficient experimental support were considered in this step because they may have support based on orthology. We lifted coordinates of exon boundaries over to the human genome assembly (hg38) using the LiftOver tool from UCSC Genome Browser [13] for all species. This allowed us to use the hg38 coordinate system as a reference to identify 1:1:1:1:1 orthologous exons across all five species, i.e., there were unique reciprocal alignments of exons. To identify orthologous alternative splicing events, we searched the coordinates of the intron chains across groups of species, limiting to alternative splicing events within the same category. An alternative splicing event was considered orthologous among a group if it was present in all species in the group. We considered orthology at four phylogenetic levels, including 17,639 orthologous events in bovida (cattle and sheep), 8961 in artiodactyla (cattle, sheep and pigs), 5352 in mammals (cattle, sheep, pigs, and horses), and 3276 in vertebrates (all fives species) (Table 4). The most abundant type of conservative alternative splicing events is the skipped exon (SEs). Importantly, we found the integration of SRA data to vastly improve the identification of conserved alternative splicing events (Table 4).

**Table 4:** Summary of conserved alternative splicing events

Lineage	Ensembl + RefSeq annotations / assembled transcripts / all SRA data					
	A5SS	SE	MXE	RI	A3SS	Total
Vertebrate	0/20/21	11/79/3126	0/7/97	0/0/0	0/30/32	11/136/3276
Mammal	0/40/42	14/140/4927	0/9/272	0/9/9	0/92/102	14/290/5352
Artiodactyla	0/17/22	5/47/8038	1/2/840	0/6/6	0/50/55	6/122/8961
Bovidae	0/47/85	13/159/14,660	0/7/2606	1/48/51	4/181/237	18/442/17,639

## Utility and discussion

**Fig. 1:**
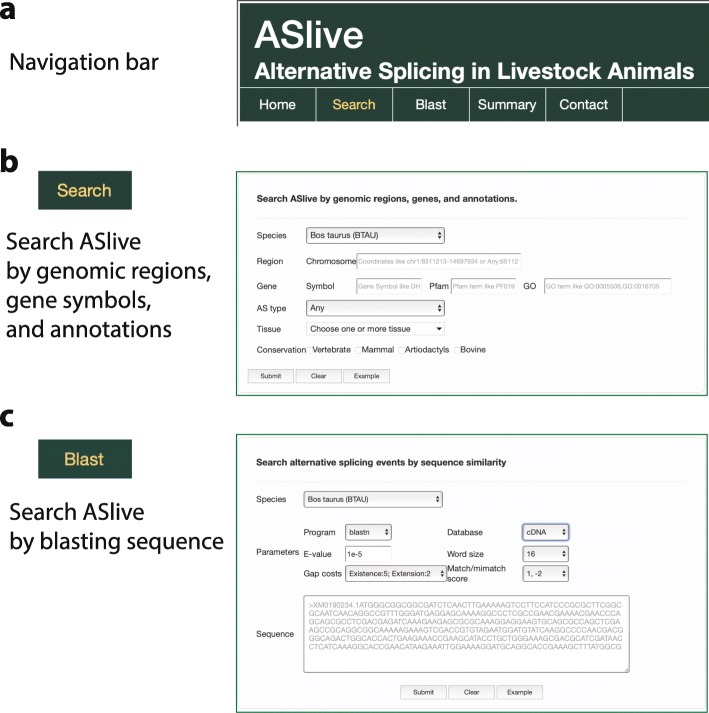
Web interface of ASlive. **a** Navigation bar of the web interface for ASlive.org. **b** Entry point for the database by search based on genomic locations, gene symbols, and annotations. **c** Entry point for the database by search based on sequence similarity

**Fig. 2:**
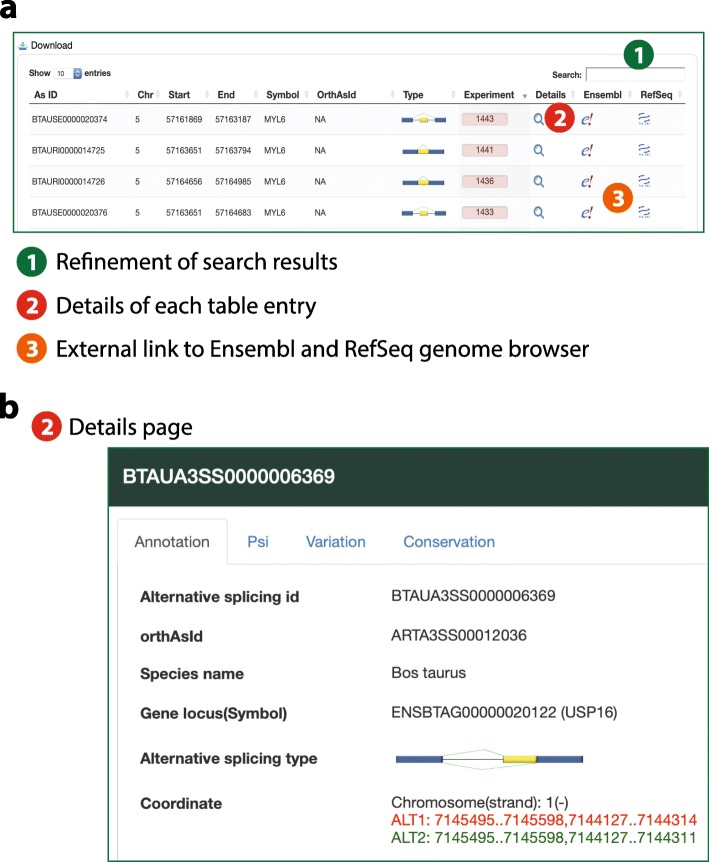
Information and data contained in ASlive. **a** Display of search results and links to additional information in ASlive. **b** Basic information on alternative splicing events and tabs in the details page that leads to additional information including PSI, overlap with DNA variation, and conservation

A simple and intuitive web interface (ASlive.org) was designed for users to explore the ASlive database (Fig. 1a). There are two primary ways to initiate a query against the database, which are easily accessible within a navigation bar of the ASlive website (Fig. 1a). Users may search the database by entering the specific genomic locations, gene symbols, or Pfam and GO annotations (Fig. 1b). Alternatively, the database can be queried by blasting a sequence (Fig. 1c). This is particularly useful when looking for orthologous genes in a different species when they are not easily identified by gene symbols. Both entry points lead to similarly structured list of alternative splicing events that match the query. The results of the search are displayed in a concise table form (Fig. 2a).

The table (Fig. 2a) can be downloaded for further analyses by the users. Within the table, users may refine the research results by imposing additional search criteria, open a pop-up window to explore the details of the alternative splicing events (also provided as a hyperlink at the AS ID), and link to the a genome browser in the context of gene models and reference annotations.

**Fig. 3:**
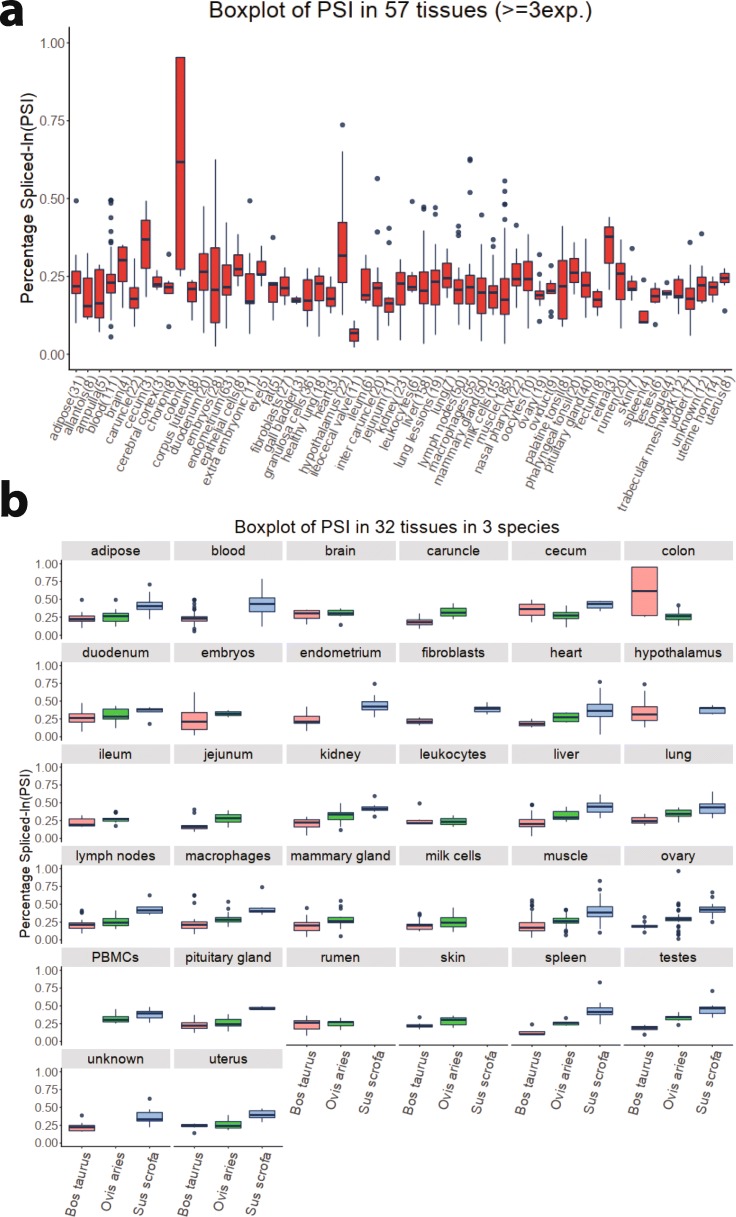
Visualization of quantitative alternative splicing information across tissues and species. Boxplots are used to display the variation within and across 57 tissues of an alternative splicing event in bovine (**a**) and the same information in 32 tissues in three species for the same event (**b**)

The details window for each alternative splicing event contains a wealth of information we gathered from either the SRA data or other databases. There are four tabs in this window. First, the annotation tab provides basic information for the event including unique ID, orthologous ID if available, classification of the event, the coordinates of exon boundaries that are involved in the splicing, and a link to a genome browser implemented in JBrowse (Fig. 2b). Second, the PSI tab offers the PSI data across all SRA experiments with tissue annotation in a sortable Table. A box plot showing the variation across experiments and tissues is also displayed (Fig. 3a). Third, the variation tab provides a list of dbSNP variants that overlap within the exons and introns of the alternative splicing event, including whether they overlap with the acceptor/donor sites. Finally, the conservation tab provides a boxplot visualization of PSIs across species where the event is conserved (Fig. 3b). These data visualizations allow users to quickly assess the biological significance of an alternatives splicing event, such as whether it is conserved or specific across tissues and species. Users may also download data associated with these visualizations to explore further details.

As RNASeq data in data archives grow, we plan to regularly update the database with new data. Our ID system of alternative splicing events allows us to add new events without altering existing IDs, providing backward compatibility. Nevertheless, the existing data already have a comprehensive coverage of tissues, cell types, and biological conditions and likely will serve most purposes. Because of the important role of genetic variation in animal related research, we plan to incorporate additional data sources that can capture the relationship among genetic variation at the DNA, splicing, and phenotypic levels. This could be, for example, achieved by incorporating genotype-phenotype associations present in the animal QTLdb (https://www.animalgenome.org) [14].

## Conclusions

We describe the development and implementation of a comprehensive alternative splicing database in livestock animals - ASlive.org. The database fills an important gap in the current literature and web space and has several unique features. First, it is the first database specifically designed for livestock animals to capture alternative splicing events in heterogeneous samples, which allows users to obtain experimental support of alternative splicing events from a wide range of tissues, cell types, and biological conditions. Unlike many other alternatives splicing databases which relies on a good assembly (typically in GTF format) to identify alternative splicing events, we used rMATs to also identify novel events that are independent of transcript assemblies. Second, we design the interface to meet various needs, including experimental biologists who focus on the details of a small number of genes or computational scientists who are interested in downloading the primary data and processing them offline. Third, we present one of the first databases to include orthologous alternative splicing events, which cannot be easily accessed through existing genome browsers and databases.

## Supplementary information

**Additional file 1:** SRA data used in the present study.

## Data Availability

All primary data are available from the SRA database and our database (http://aslive.org) is fully open. SRA accessions used in study are listed in Additional file 1.

## Abbreviations

A3SS: Alternative 3′ splice site
A5SS: Alternative 5′ splice site
MXE: Mutually exclusive exon
PSI: Percent spliced in
RI: Retained intron
SE: Skipped exon
SRA: Sequence read archive
